# Gaze behavior in open-angle glaucoma patients during visuo-cognitive-motor tasks: a cross-sectional study

**DOI:** 10.1038/s41598-024-70987-2

**Published:** 2024-09-09

**Authors:** Constantin W. Freitag, Martin Behrens, Robert Bielitzki, Khaldoon O. Al-Nosairy, Francie H. Stolle, Gokulraj T. Prabhakaran, Rosalie Beyer, Hagen Thieme, Michael B. Hoffmann, Lutz Schega

**Affiliations:** 1 Department of Sport Science, Institute III, Otto-von-Guericke University Magdeburg, Zschokkestraße 32, 39104 Magdeburg, Germany; 2 University of Applied Sciences for Sport and Management Potsdam, Olympischer Weg 7, 14471 Potsdam, Germany; 3 Ophthalmic Department, University Hospital Magdeburg, Leipziger Str. 44, 39120 Magdeburg, Germany; 4 Center for Behavioral Brain Research, Magdeburg, Germany

**Keywords:** Diagnostic markers, Visual system

## Abstract

This study investigated gaze behavior during visuo-cognitive-motor tasks with a change of movement direction in glaucoma patients and healthy controls. Nineteen glaucoma patients (10 females, 9 males) and 30 healthy sighted controls (17 females, 13 males) participated in this cross-sectional study. Participants performed two visuo-cognitive-motor tasks with a change of movement direction: (i) the “Speed-Court-Test” that involved stepping on different sensors in response to a visual sign displayed on either a large or small screen (165″ and 55″, respectively); (ii) the “Trail-Walking-Test” that required walking to 15 cones labeled with numbers (1–8) or letters (A-G) in an alternately ascending order. During these tasks, the time needed for completing each task was determined and the gaze behavior (e.g., saccade duration, fixation duration) was recorded via eye tracking. Data were analyzed with repeated measures analyses of covariance (ANCOVA; GROUP × SCREEN) and one-way ANCOVA. No differences between groups were found for the time needed to complete the tasks. However, during the “Trail-Walking-Test”, the fixation duration was longer for glaucoma patients than for controls (p = 0.016, ${\upeta }_{\text{p}}^{2}$ = 0.131). Furthermore, during the “Speed-Court-Test”, there was a screen size effect. Irrespective of group, saccade amplitudes were lower (p < 0.001, ${\upeta }_{\text{p}}^{2}$ = 0.242) and fixation durations were higher (p = 0.021, ${\upeta }_{\text{p}}^{2}$ = 0.125) for the small screen. Fixation durations were longer in glaucoma patients during the cognitively demanding “Trail-Walking-Test”, which might indicate a strategy to compensate for their visual impairment.

## Introduction

Glaucoma belongs to a group of slowly progressing optic neuropathies^1^. It is considered the most common cause of irreversible blindness world wide^2^ with 76 million people affected in 2020 and forecasted to increase up to 111.8 million by 2040^3^.

Glaucoma is characterized by a thinning of the retinal ganglion cell layer due to axonal degeneration of the retinal ganglion cells^4^. It usually leads to peripheral visual field loss, which is almost unnoticeable in the early stages of glaucoma^5^, but its extent increases with disease duration^1,3, 6^. The retinal ganglion cell decay affects great parts of the post-retinal visual system including subcortical regions^4,7, 8^. Preperimetric glaucoma patients have been shown to exhibit altered patterns of saccadic eye movements in comparison to healthy sighted controls^9^, with a probable involvement of cortical and subcortical structures^8^ including those related to the control of saccadic eye movements^10^. Consequently, alterations of saccadic eye movements might serve as a biomarker for altered subcortical retinal projections^11^.

These glaucoma-induced changes might impair activities of daily living, such as reading, walking, and driving^11,12^. However, studies addressing the gaze behavior of glaucoma patients reported heterogenous results^13,14^. Very few previous studies have assessed gaze behavior in glaucoma patients in comparison to healthy sighted controls while walking through an environment depicting a real-world condition to examine potential compensatory strategies during activities of daily living^11,15^. In a real-life shopping task^16,^ the authors reported that glaucoma patients glanced frequently towards their visual defect area^16^. Furthermore, Cheong et al. conducted a real-life street crossing task^13^ and reported a reduced fixation area and a different fixation distribution in the glaucoma patients compared with the healthy sighted controls, which is in contrast to Geruschat et al.^17^, who failed to find differences in the number of fixations between glaucoma patients and healthy sighted controls. However, these differences might be related to the different tasks (no street crossing^13^ vs. street crossing^17^ street crossing). In another study by Lajoie et al. glaucoma patients and healthy sighted controls walked through an obstacle navigation course during single- and dual-task conditions, respectively^18^. The authors demonstrated increased fixation durations and fixation counts for the single-task condition in glaucoma patients^18^. Nevertheless, none of these studies recorded gaze behavior during visuo-cognitive-motor tasks that require walking with a change of movement direction.

In addition to visual deficits, it has previously been discussed that glaucoma might also be related to cognitive impairments^19^. Therefore, the present study aimed to investigate differences in execution time and gaze behavior during two different visuo-cognitive-motor tasks between glaucoma patients and healthy sighted controls. For this purpose, the interactive Speed Court^®^ system (GlobalSpeed GmbH, Hemsbach, Germany)^20,21^, here referred to as “Speed-Court-Test” (primary outcome), and the “Trail-Walking-Test”^22^ (secondary outcome) were used to examine gaze behavior, cognitive performance, and functional mobility. These tasks demand gaze shifts to (“Speed-Court-Test”) or search for a visual target (“Trail-Walking-Test”), making a visual stimulus-dependent decision, and a corresponding motor action with a change of movement direction.

It was hypothesized that (i) glaucoma patients have longer execution times during the “Speed-Court-Test” and the “Trail-Walking-Test” compared to healthy sighted controls. Moreover, based on the study by Lajoie et al.^18^, it was assumed that (ii) glaucoma patients have longer fixation durations^18^ compared to healthy sighted controls during the “Speed-Court-Test” and the “Trail Walking-Test”.

## Methods

### Study design

In this article, the presented data were recorded during the baseline measurements of a longitudinal study examining the effects of a multimodal vs. unimodal exercise intervention on visual, motor, and cognitive performance measures as well as structural and functional brain adaptations in glaucoma patients and healthy sighted control participants (German Clinical Trial Register, ID: DRKS00022519/05.08.2020, https://drks.de/search/de/trial/DRKS00022519). The study was approved by the Ethics Committee of the University Medical Faculty Magdeburg (32/18) and performed in accordance with the tenets of the Declaration of Helsinki. All participants signed the informed consent form before participating in this study.

### Participants

A sample size calculation for a repeated measures analysis of variance (ANOVA) with two groups (GROUP: glaucoma patients, healthy sighted controls) and two conditions (small screen and large screen) for the “Speed-Court-Test” was performed with G*Power (version 3.1)^23^. The following input variables were used: medium effect size (f = 0.25), a significance level of 0.05 with a power of 0.95 and correlation among the repeated measures of 0.7. According to the calculation, a total sample size of 34 participants (17 participants per group) was required.

All glaucoma and healthy sighted participants underwent standard ophthalmological examination including best corrected visual acuity (BCVA) and visual field (VF), and optical coherence tomography (OCT). For BCVA testing, early treatment of diabetic retinopathy standard (ETDRS) charts were used. For VF testing, standard automated perimetry employing the Swedish Interactive Threshold Algorithm 24-2 protocol (SITA-Fast) of the Humphrey Field Analyzer 3 (Carl Zeiss Meditec AG, Jena, Germany) was used for testing each eye separately. For binocular VF testing, a 24-2 binocular VF testing was conducted using the Ocusweep™ Perimeter (Ocusweep SAP, Ocuspecto Ltd, Turku, Finland). Ocusweep perimeter enabled VF testing without head or chin rest under ambient light conditions. For both VF tests, the mean deviation (MD [dB]) from a control cohort was determined for VF sensitivity assessment, where negative values indicate greater VF loss. For structural data assessment, OCT scans were acquired using a spectral domain OCT device employing Glaucoma Module Premium edition (Heidelberg Spectralis®, Heidelberg Engineering, Heidelberg, Germany) to estimate peripapillary retinal nerve fiber layer thickness (pRNFL) of each eye. After ophthalmological examination, glaucoma was defined as follows: (i) optic disc cupping with a vertical cup-to-disc ratio ≥ 0.7, (ii) retinal fiber layer defect and/or a local notching of the optic disc rim, and/or (iii) characteristic glaucomatous VF defects. All patients were on IOP-lowering medication.

The recruitment of the participants took place at the Department of Ophthalmology at the University Hospital Magdeburg, via local ophthalmologists and national patient networks. The following inclusion criteria were applied: (i) ≥ 60 years, (ii) diagnosis of open-angle glaucoma (only for the glaucoma group), (iii) BCVA ≥ 0.8 decimal unless glaucoma-related in the OAG group, (iv) normal visual function parameters unless glaucoma-related, (v) no other conditions affecting visual function, and (vi) ability to walk at least 6 min without walking support. The exclusion criteria were as follows: (i) any eye disease affecting the visual acuity or VF estimates of visual function (e.g., cataract [except incipient stage], ocular trauma history and ocular surgeries [except glaucoma or cataract surgery]), (ii) neurological diseases, and (iii) conditions that limit the physical performance of the participants (orthopedic diseases including arthrosis (grade II or higher), musculoskeletal impairments, tendinitis, tenosynovitis, myositis, prosthesis in the lower extremities, joint replacements, neurological disorders, rheumatism, cardiovascular disorders, stroke, and/or heart-rate related disease).

Overall, 49 participants, 19 with glaucoma (glaucoma group: 9 males, 10 females, [mean ± standard deviation], age 71 ± 6 years, height 168.7 ± 8.5 cm, body mass 73.0 ± 16.7 kg) and 30 age-matched healthy sighted participants (control group: 13 males, 17 females, age 71 ± 5 years, height 168.3 ± 10.4 cm, body mass 75.3 ± 17.8 kg) were included in this study (see Table 1 for ophthalmological details).

**Table 1:** Ophthalmological characteristics of the participants.

	Controls (n = 30)	Glaucoma (n = 19)	Difference
	Median|range	Median|range	p-value
MD_right	0.59|5.09	− 0.67|25.97	0.015
MD_left	0.15|5.92	− 1.09|22.00	0.009
MD_binocular	0.70|3.3	− 0.40|9.6	< 0.001

	Mean ± SD	mean ± SD	p-value
BCVA	− 0.11 ± 0.08	− 0.07 ± 0.13	0.236
pRNFL_right	91 ± 12.29	77 ± 12.71	< 0.001
pRNFL_left	89 ± 10.81	76 ± 14.94	< 0.001
IOP_right	15.7 ± 2.8	16.9 ± 5.9	0.435
IOP_left	16.5 ± 3.8	16.9 ± 6.1	0.773
SE_binocular	0.8 ± 1.5	0.2 ± 1.8	0.227

BCVA: best corrected visual acuity [logMAR], pRNFL_right/left: peripapillary retinal nerve fiber layer (pRNFL) thickness [µm], MD_right/left: mean deviation [dB], IOP: intraocular pressure, SE: spherical equivalent,SD: standard deviation.

### General procedure

Over a period of two consecutive days (at the same time of day), the participants performed several tests including single- and dual-task gait analyses and two visuo-cognitive-motor tasks: the “Speed Court-Test” and the “Trail-Walking-Test”^22^.

At the beginning of day one, each participant underwent the following procedure: (i) signing the informed consent, (ii) documenting their physical activity with the German version of the Freiburger Questionnaire on Physical activity^24^, (iii) measuring anthropometric data, (iv) assessing static postural control using the MFT S3 check (MFT Bodyteamwork GmbH, Kirchberg, Austria)^25^, (v) single-task walking, (vi) dual-task walking, and (vii) visuo-cognitive-motor task performance. On day two, the participants underwent the following procedure: (i) single-task walking, (ii) dual-task walking and (iii) visuo-cognitive-motor task. The visuo-cognitive-motor tasks and the dual-task gait test were conducted in a randomized order. In this regard, one of the visuo-cognitive-motor tasks was performed on the first day and the other on the second day. Due to the COVID-19 pandemic, participants were instructed to wear an FFP2 mask during all laboratory visits.

### Gaze behavior data

Participants were equipped with mobile binocular eye tracking glasses (Senso-Motoric Instruments, SMI ETG 2, Teltow, Germany, resolution of 1280 × 960 pixels, sampling frequency of 60 Hz). The eye tracker's gaze position accuracy was indicated by the manufacturer to be of 0.5°^26^. Precision values of the mobile system were not provided by the manufacturer. Pastel^26^ and Hooge^27^ reported precision values ranging from 0.03° to 0.32°. A Samsung Galaxy S4 Smartphone (Samsung, Seoul, South Corea, 1.7 GHz processor) with the iView ETG Software (Senso-Motoric Instruments, Teltow, Germany) was used to record the data for later analysis. At the beginning of each measurement session, an instant cursor three-point calibration was conducted according to the manufacturer’s calibration protocol. To compensate for refractive error, the mobile eye tracker was equipped with lenses matching the spherical equivalent of refractive correction when required. The recorded data were analyzed with the BeGaze software, using the SMI Event Detection algorithm (Senso-Motoric Instruments, Version 3.7.40, Teltow, Germany). Eye-tracking data before the start and after the end of the visuo-cognitive-motor tasks were excluded. Outcome variables of interest were performance (s), saccade duration (ms), saccade frequency (saccades/s), saccade amplitude (°), fixation duration (ms), fixation frequency (fixations/s).

### Visuo-cognitive-motor tasks

The visuo-cognitive-motor tasks included the “Speed-Court-Test” and “Trail-Walking-Test”^22^.

**Fig. 1:**
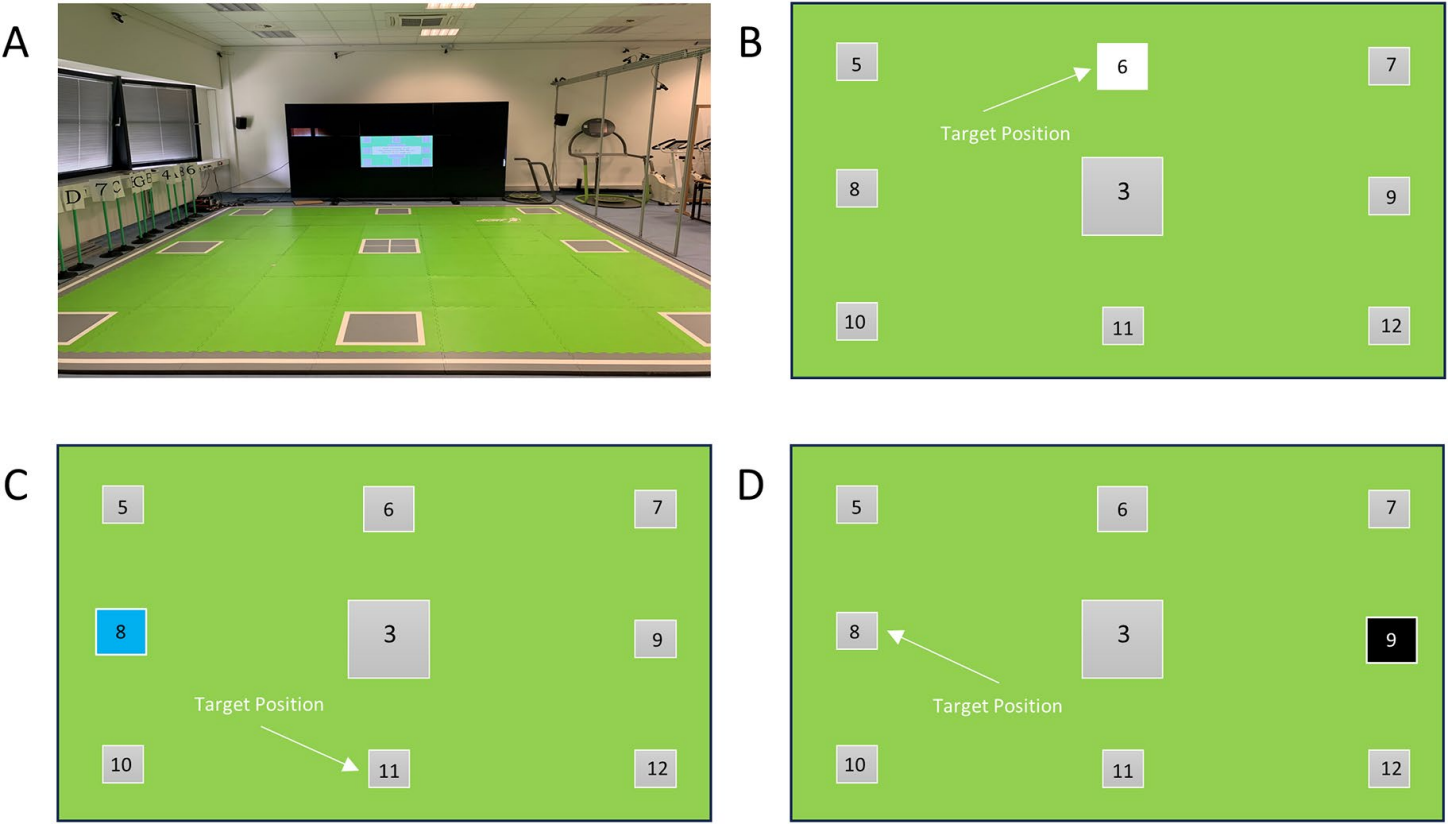
Experimental set-up of the “Speed-Court-Test”, condition small screen (**A**). Explanation of the color specific stimuli: white stimulus, participant had to go the displayed sensor plate (**B**); blue stimulus, participant had to go to sensor plate 11 (**C**); black stimulus, participant had to go to the opposite sensor plate (**D**).

The “Speed-Court-Test” was conducted using the interactive system Speed Court® (GlobalSpeed GmbH, Hemsbach, Germany)^20^. In the context of the present study, Speed-Court was used to assess cognitive flexibility^28^ and functional mobility. The Speed Court® System consists of an area (5 × 5 m) with 12 sensor plates placed on the floor, which are connected to a computer. Using the manufacturers software, the sensors can be displayed on a screen and the participants had to step on specific sensors depending on which was shown on the screen (see Fig. 1). One test trial included 36 randomized colored (white, blue, black) stimuli, which were shown on a display wall consisting of 9 monitors, three per row (55″ screen size, resolution of 1920 × 1080 pixel, respectively). Each color was assigned to a specific task: (i) white stimulus: participant had to go the displayed sensor plate; (ii) blue stimulus: participant had to go to sensor plate 11, and (iii) black stimulus: participant had to go to the opposite sensor plate (see Fig. 1). After the presentation of each stimulus, the participants had to go to the center plate before the next stimulus was presented. The test included two screen conditions (see Fig. 1): (i) large screen, 165″ (height: 205.2 cm, width: 364.2 cm) and (ii) small screen, 55″ (height: 68.4 cm, width: 121.4 cm, center monitor of the display wall), which the participants had to perform three times in a randomized order, respectively. Approximate minimal and maximal viewing distances were 1 m and 5.5 m, respectively. The participants performed one familiarization trial for each screen condition, followed by the experimental trials. The starting position was behind the center sensor plate with the eyes directed to the display wall. The participants triggered the 3-s countdown for the start of the test by tipping on the center sensor plate. The time to accomplish the task (“performance”) and the eye tracking data of the fastest trial for each screen condition (large screen and small screen condition) were analyzed.

**Fig. 2:**
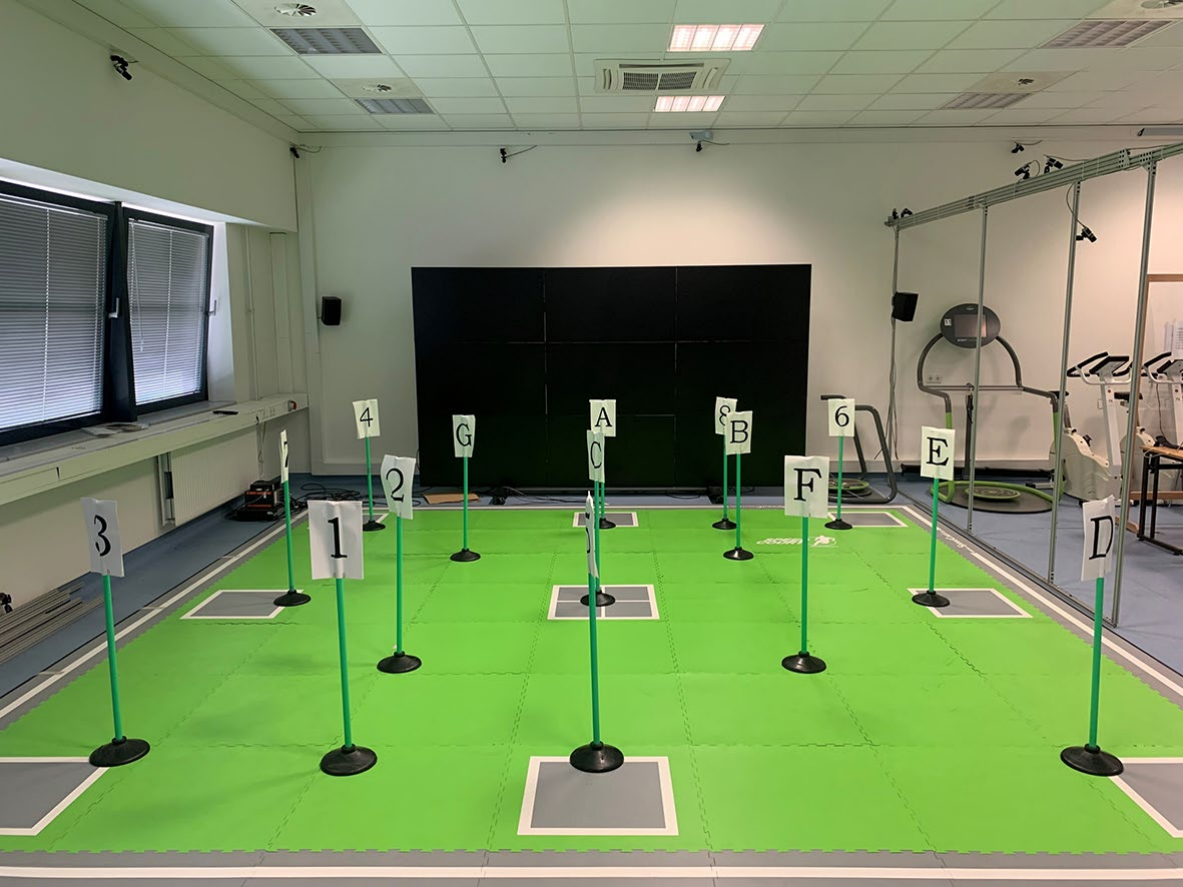
Experimental set-up of the “Trail-Walking-Test”. The participants had to walk towards the cones in an alternately ascending order (1, A, 2, B, 3, C and so on).

The participants conducted the modified “Trail-Walking-Test” (third condition of the Trail-Walking-Test by Schott^22)^ to asses working memory, cognitive flexibility, and functional mobility. The investigator positioned 15 cones, labeled with numbers or letters, on a 5 × 5 m large area. Each cone, depending on the number (1 to 8) or letter (A to G) had a designated fixed position (see Fig. 2). The participants were instructed to walk towards the cones in an alternately ascending order (1, A, 2, B, 3, C and so on) as fast as possible and safe as possible (without running). They had to touch each cone with one foot to signal the decision to the instructor before walking to the next cone. If the wrong cone was selected (e.g., 2, C), the investigator indicated: “STOP, please go back to the last cone”. The starting position was next to the cone with the number 1. After the start signal, the time was recorded until the cone with the number 8 was touched. The participants performed the modified “Trail-Walking-Test” three times and the time required to accomplish the task, i.e. performance, as well as the eye tracking data of the fastest trial were used for analyses.

### Statistical analysis

Data were analyzed using the Statistical Package for Social Science (SPSS® Statistics Version 28.0, IBM^®^ Corp., New York, USA). Normal distribution was checked using the Shapiro–Wilk test. Since previous studies have shown the repeated measures ANCOVA and independent t-tests to be robust against moderate violation of normality^29–31^, nonparametric tests were not used to check for differences. Differences were considered significant when *p* ≤ 0.05.

Differences between the groups in the anthropometric data were checked with independent t-tests. To analyze the data recorded during the “Speed-Court-Test”, a two-way repeated measures ANCOVA with the between-subject factor GROUP (glaucoma patients versus healthy sighted controls) and within-subject factor SCREEN (large screen and small screen condition) was performed to compare the data of groups. Differences between groups regarding the data recorded during the “Trail-Walking-Test” were checked using a one-way ANCOVA. Given that the number of males and females differed between groups, sex was used as covariate. In case of significant main or interaction effects, Bonferroni-corrected post-hoc tests were conducted. Data are presented as means ± standard deviations and mean differences with 95% confidence intervals (CI). The effect size partial eta squared (${\upeta }_{\text{p}}^{2}$) was calculated for the ANCOVA and was interpreted according to Lakens^32^: small (${\upeta }_{\text{p}}^{2}$ ≥ 0.01), medium (${\upeta }_{\text{p}}^{2}$ ≥ 0.06), and large effect (${\upeta }_{\text{p}}^{2}$ ≥ 0.14). Additionally, if main effects were detected, a secondary analysis (two-way repeated measures ANCOVA or one-way ANCOVA) excluding partcipants with adavanced  glaucoma was conducted to check if the severity of the glaucoma stage has an impact on the present results.

Further, correlations between gaze behavior, visuo-cognitive motor task performance and visual performance in glaucoma patients were examined. For normal distributed data, the Pearson correlation or for non-normal distributed data the Spearman correlations coefficient was used. The correlations coefficients were interpreted according Cohen^33^: small (r ≥ 0.1), medium (r ≥ 0.3), and large (r ≥ 0.5). Due to the explorative nature of the analysis in the present study, no correction for multiple testing was applied.

## Results

**Fig. 3:**
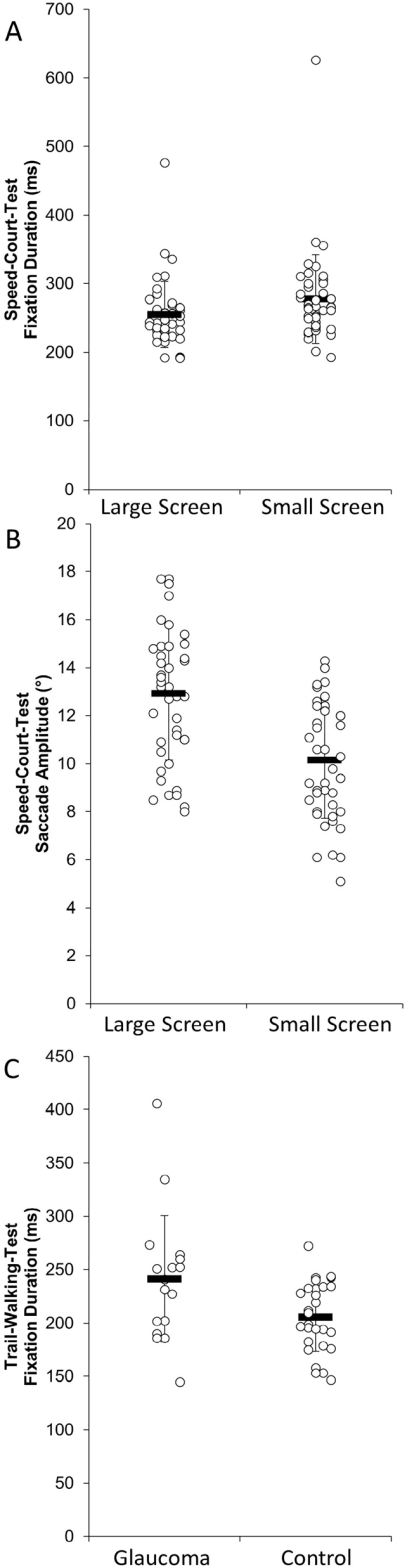
Means ± standard deviations and individual data for the fixation duration (**A [**p = 0.021**]**) and the saccade amplitude (**B** [p < 0.001]) during the “Speed-Court-Test” of all participants for the condition large screen (n = 49) and small screen (n = 49), and the fixation duration (C [p = 0.016]) during the “Trail-Walking-Test” of the glaucoma patients (n = 17) and healthy sighted controls (n = 28).

Due to invalid data and drop-outs, data of nine participants (six of the control group and three of the glaucoma group) were not completely included in the analyses. Furthermore, 2 participants with advanced glaucoma were excluded only for the secondary analysis. To increase transparency, the number of analyzed cases for the respective parameter is shown in Tables 2, 3. An overview of all parameters including the p-values and effect sizes is presented Tables 2, 3 and in Fig. 3.

**Table 2:** Means ± standard deviations of the eye tracking data during the “Speed-Court-Test” of glaucoma patients and healthy sighted controls as well as the outcomes of the ANCOVA.

Condition	Large Screen	Small Screen	Repeated Measures ANCOVA		
			Screen	Group	Screen × Group
Performance (s)					
Control N = 27	91.590 ± 15.325	89.43 ± 15.667	F(1,40) = 0.077 p = 0.782 ${\upeta }_{\text{p}}^{2}$ = 0.002	F(1,40) = 0.455 p = 0.504 ${\upeta }_{\text{p}}^{2}$ = 0.011	F(1,40) = 2.757 p = 0.105 ${\upeta }_{\text{p}}^{2}$ = 0.064
Glaucoma N = 16	86.550 ± 10.993	88.143 ± 13.947			
Saccade Duration (ms)					
Control N = 27	154.341 ± 39.113	136.193 ± 40.639	F(1,40) = 1.462 p = 0.234 ${\upeta }_{\text{p}}^{2}$ = 0.035	F(1,40) = 1.282 p = 0.264 ${\upeta }_{\text{p}}^{2}$ = 0.031	F(1,40) = 0.669 p = 0.418 ${\upeta }_{\text{p}}^{2}$ = 0.016
Glaucoma N = 16	146.749 ± 27.504	123.669 ± 23.765			
Saccade Frequency (saccades/s)					
Control N = 27	1.696 ± 0.384	1.641 ± 0.313	F(1,40) = 0.436 p = 0.513 ${\upeta }_{\text{p}}^{2}$ = 0.011	F(1,40) = 0.268 p = 0.608 ${\upeta }_{\text{p}}^{2}$ = 0.007	F(1,40) = 0.011 p = 0.919 ${\upeta }_{\text{p}}^{2}$ = 0.000
Glaucoma N = 16	1.744 ± 0.354	1.694 ± 0.386			
Saccade Amplitude (°)					
Control N = 27	13.415 ± 2.312	10.448 ± 2.353	**F(1,40) = 12.742** **p < 0.001** ${{\varvec{\upeta}}}_{\mathbf{p}}^{2}$ **= 0.242**	F(1,40) = 2.210 p = 0.145 ${\upeta }_{\text{p}}^{2}$= 0.052	F(1,40) = 1.001 p = 0.323 ${\upeta }_{\text{p}}^{2}$ = 0.024
Glaucoma N = 16	12.119 ± 3.453	9.706 ± 2.680			
Fixation Duration (ms)					
Control N = 27	246.807 ± 31.467	269.241 ± 36.793	**F(1,40) = 5.272** **p = 0.021** ${{\varvec{\upeta}}}_{\mathbf{p}}^{2}$ **= 0.125**	F(1,40) = 1.506 p = 0.227 ${\upeta }_{\text{p}}^{2}$ = 0.036	F(1,40) = 0.000 p = 0.993 ${\upeta }_{\text{p}}^{2}$ = 0.000
Glaucoma N = 16	269.188 ± 69.902	292.131 ± 97.378			
Fixation Frequency (fixation/s)					
Control N = 27	2.1 ± 0.253	2.063 ± 0.204	F(1,40) = 2.103 p = 0.155 ${\upeta }_{\text{p}}^{2}$ = 0.05	F(1,40) = 0.466 p = 0.499 ${\upeta }_{\text{p}}^{2}$ = 0.12	F(1,40) = 0.049 p = 0.827 ${\upeta }_{\text{p}}^{2}$ = 0.001
Glaucoma N = 16	2.144 ± 0.299	2.094 ± 0.317			

Significant differences are presented in bold.

**Table 3:** Means ± standard deviations of the eye tracking data during the “Trail-Walking-Test” of glaucoma patients and healthy sighted controls as well as the outcomes of the ANCOVA.

		One-way ANCOVA
		Group
Performance (s)		
Control N = 28	56.457 ± 13.664	F(1,42) = 0.416 p = 0.522, ${\upeta }_{\text{p}}^{2}$ = 0.010
Glaucoma N = 17	59.440 ± 14.384	
Saccade Duration (ms)		
Control N = 28	173.882 ± 41.612	F(1,42) = 3.320 p = 0.076, ${\upeta }_{\text{p}}^{2}$ 0.073
Glaucoma N = 17	152.847 ± 35.770	
Saccade Frequency (saccades/s)		
Control N = 28	1.811 ± 0.511	F(1,42) = 0.120 p = 0.730, ${\upeta }_{\text{p}}^{2}$ = 0.003
Glaucoma N = 17	1.759 ± 0.433	
Saccades Amplitude (°)		
Control N = 27	12.186 ± 2.287	F(1,42) = 1.365 p = 0.249, ${\upeta }_{\text{p}}^{2}$ = 0.031
Glaucoma N = 16	11.335 ± 2.907	
Fixation Duration (ms)		
Control N = 28	205.921 ± 32.859	**F(1,42) = 6.331** **p = 0.016,** ${{\varvec{\upeta}}}_{\mathbf{p}}^{2}$** = 0.131**
Glaucoma N = 17	241.465 ± 60.885	
Fixation Frequency (fixation/s)		
Control N = 27	2.164 ± 0.352	F(1,42) = 0.088 p = 0.768, ${\upeta }_{\text{p}}^{2}$ = 0.002
Glaucoma N = 16	2.188 ± 0.264	

Significant differences are presented in bold.

A significant SCREEN effect was found for fixation duration during the “Speed-Court-Test” (F (1,40) = 5.272, p = 0.021; ${\upeta }_{\text{p}}^{2}$ = 0.125). The post-hoc test indicated that the fixation duration was longer in the small screen compared to the large screen condition, irrespective of the group (p < 0.001). The secondary analysis revealed also a SCREEN effect (F (1,38) = 5.453, p = 0.025, ${\upeta }_{\text{p}}^{2}$ = 0.125). The post-hoc test indicated that the fixation duration was longer in the small screen compared to the large screen condition, irrespective of the group (p < 0.001). Furthermore, a significant SCREEN effect for saccade amplitude during the “Speed-Court-Test” was found (F (1,40) = 12.742, p < 0.001, ${\upeta }_{\text{p}}^{2}$ = 0.242). The post-hoc test indicated saccade amplitudes to be smaller for the small screen compared to the large screen condition, irrespective of the group (p < 0.001), see Fig. 3 and Table 2. The secondary analysis revealed also a SCREEN effect (F (1,38) = 12.617 p = 0.001, ${\upeta }_{\text{p}}^{2}$ = 0.249). The post-hoc test also indicated saccade amplitudes to be smaller for the small screen compared to the large screen condition, irrespective of the group (p < 0.001).

For the “Trail-Walking-Test” a significant GROUP effect was evident for fixation duration (F (1,42) = 6.331, p = 0.016, ${\upeta }_{\text{p}}^{2}$ = 0.131, mean difference = 35.536, 95% CI = 7.035—64.038). The fixation durations were longer in the glaucoma group than for the controls (see Fig. 3 and Table 3). The secondary analysis revealed also a GROUP effect (F (1,40) = 5.330, p = 0.026, ${\upeta }_{\text{p}}^{2}$ = 0.118, mean difference = 34.635, 95% CI = 4.315–64.955).

No significant correlations between gaze behavior, visuo-cognitive motor-task performance and visual performance (binocular VF mean deviation, better eye VF mean deviation, better eye pRNFL, better eye visual acuity, better eye dynamic visual acuity) in glaucoma patients were found.

## Discussion

To the authors’ knowledge, the present study is the first investigating gaze behavior during visuo-cognitive-motor tasks with a change of direction in glaucoma patients and healthy sighted controls. The main findings were: (i) longer fixation durations in glaucoma patients during the “Trail-Walking-Test” and (ii) irrespective of group, longer fixation durations and smaller saccade amplitudes in the small- compared with large-screen condition during the “Speed-Court-Test”.

The findings from the present study revealed longer fixation durations during the “Trail-Walking-Test” in glaucoma patients compared with healthy sighted controls. Several studies reported conflicting results regarding the gaze behavior of glaucoma patients. For instance, glaucoma patients have been found to show a higher saccade and fixation frequency as well as shorter fixation duration compared to healthy sighted controls when watching driving scenes^14^. This was interpreted as a strategy to compensate for their VF loss. In contrast, the results of the real-world street crossing study from Cheong et al. indicate no difference between glaucoma patients and healthy sighted controls for saccades, but the authors have found a reduced fixation area and a different fixation distribution for the glaucoma patients^13^. The authors explained their findings with a reduction of the visual scanning area, which might have resulted in missing peripheral vision information during walking^13^. In accordance with the results of the present study, Lajoie et al. have also found that glaucoma patients showed a longer fixation duration while negotiating obstacles (vertical pole)^18^. This might be related to the fact that peripheral VF loss can cause glaucoma patients to rely more on central vision^18^. Of note, in the present study, a non-significant trend towards shorter saccade durations during the “Trail-Walking-Test” in glaucoma patients compared with healthy sighted controls was found (*p* = *0.076,* ${\eta }_{p}^{2}$ = *0.073*). The longer fixation duration and the lower time to look for the next cone in advance might be a strategy to compensate for the VF loss^18,34^. Interestingly, despite these differences in the gaze behavior between groups, performance during the “Trail-Walking-Test” was not different between glaucoma patients and healthy sighted controls.

Furthermore, there were no between-group differences for the test performance and gaze behavior during the “Speed-Court-Test”, regardless of the factor SCREEN. This finding is in contrast to other studies that investigated visual search tasks in which the participants were not able to move their head. It has been found that glaucoma patients showed higher saccade frequency, fixation frequency, frequency duration, and saccadic velocity compared with healthy sighted controls^14,35^. Hence, in the present study, glaucomatous peripheral VF loss might have had no impact on gaze behavior because it might be compensated by head movements. Moreover, the group similarities in test performance and gaze behavior might be attributed to the mild stage of glaucoma progression in the patients of the present study.

It was assumed that glaucoma can be associated with cognitive dysfunction^19^, however, data of a previous study by our research group revealed no differences in cognitive test performance in the same glaucoma patients compared with healthy controls. This might be an explanation for the absence of group-differences in the glaucoma patients of this study^36^. Therefore, differences in gaze behavior between glaucoma patients and healthy sighted controls might dependent on other factors than cognitive function, e.g., type of task^11^. Regarding this, while the demands of the “Speed-Court-Test” did not appear to reveal differences in gaze behavior between glaucoma patients and healthy sighted controls, the “Trail-Walking-Test” might be suitable to mirror disease progression in mild stage of glaucoma. An assumption that deserves further attention in future studies.

The following limitations have to be considered in the present study. First, the tests were performed on 2 days, due to the duration of the entire testing protocol. This is less of a concern, given that this study design was chosen to avoid state fatigue^37^ induced by the testing procedure, which has been shown to influence gaze behavior^38^. As a second limitation, the environmental lightning condition was not fully standardized affecting scatter in the eye tracking data^39^. However, the observed group effects clearly exceeded this additional noise intrusion into the data. Third, due to the explorative nature of the analysis in the present study, no correction for multiple testing was applied.

In conclusion, the present study revealed a longer fixation duration during the “Trail-Walking-Test” in glaucoma patients, which might be a strategy to compensate for the VF loss. This gaze behavior might help to gather information about the environment to decrease the risk of falls. Furthermore, the longer fixation duration might serve as a biomarker for the effect of glaucoma on motor behavior, as reflected here by the oculo-motor behavior. However, given that performance and gaze behavior during the “Speed-Court-Test” was not different between the glaucoma patients and healthy sighted controls, group-differences seem to depend on the kind of visuo-cognitive-motor tasks.

## Data Availability

Data are available on reasonable request to the corresponding author.
